# A 3D-Force and Torsion Sensor Using Patterned Color Encoding

**DOI:** 10.3390/s26051534

**Published:** 2026-02-28

**Authors:** Tak Nok Douglas Yu, Hao Ren, Yajing Shen

**Affiliations:** 1 Department of Electronics and Computer Engineering, The Hong Kong University of Science and Technology, Hong Kong, China; 2 Shenzhen Research Institute, The Hong Kong University of Science and Technology, Shenzhen 518057, China

**Keywords:** tactile sensing, 3D-force, color encoding

## Abstract

Current multi-axis force sensors often rely on complex mechanical structures or arrays of discrete transducers, resulting in larger footprints, higher complexity, and limited scalability for compact applications such as robotic fingertips or wearable tactile interfaces. To address these limitations, this paper introduces a novel optical sensing approach that uses a top-layer patterned color surface and an array of color sensors to decouple and measure normal, shear, and torsional forces within a highly compact 15 × 15 mm footprint. The patterned surface functions as a visual encoding layer, where applied forces induce measurable, direction-dependent shifts in reflected color distribution. By deploying multiple color sensors in an array, each sensor captures localized color variations, enabling spatial reconstruction of both magnitude and direction of applied loads through differential color analysis. The sensor’s performance was validated through robotic gripper integration, where it successfully provided multi-axis force feedback and enabled adaptive gripping force adjustment to achieve robust and stable object manipulation. The experimental results confirm the system’s ability to effectively sensing 3D forces and torsion forces, and support closed-loop control in adaptive robotic grasping. This design presents a scalable, low-profile alternative to conventional multi-axis force sensors, suitable for integration into space-constrained robotic and haptic systems.

## 1. Introduction

The ability to perceive and measure multi-axis contact forces, including normal pressure, shear, and torque, is a common challenge for advanced robotic systems [1]. Conventional rigid body multi-axis force sensors often rely on complex mechanical structures or dense transducer arrays [2,3]. These approaches typically result in larger sizes and limited scalability, making them not suitable for deployment in compact applications such as robotic fingertips, prosthetic limbs, or wearable haptic interfaces [4,5]. Consequently, developing a sensor that integrates high-performance multi-axis measurement, a minimal form factor, and scalable fabrication remains a critical challenge in robotics and tactile sensing research.

Recent innovations have sought to address these limitations through diverse transduction principles. Magnetic-based tactile sensors (MBTS) utilize the displacement of magnetic particles within an elastomer, measured by Hall effect sensors, to achieve compact designs with high operational frequency and robustness, suitable for real-time dynamic feedback [6,7,8,9]. A “magnetic skin” sensor by Hu et al. [10] is a promising solution, combining the advantages of MBTS with a large-area magnetic skin for multi-point and multi-scale tactile sensing. They used signal processing techniques such as a K-Nearest Neighbors (KNN) classifier and a convolutional neural network (CNN) to achieve super-resolution using an array of 4 × 4 Hall sensors over 48,400 mm^2^ and an average localization error of 1.2 mm. However, this sensor is not suitable for integration into small form factors due to the computational complexity of the CNN network and susceptibility to electromagnetic interference.

Optical tactile sensors offer another powerful method, with camera-based systems like GelSight providing exceptionally high-resolution contact geometry and texture data [4,11,12]. The GelSight system by Adelson et al. [13] uses a sensor skin made of flexible gel material that is coated with a metallic powder, and a camera is used to capture reflected light from surrounding light sources to measure the deformation of the sensor surface. The surface can then be reconstructed using a 3D reconstruction algorithm, providing a high-resolution representation of the surface. Nevertheless, this method is generally more bulky due to the need for a full camera system and the system also produces too much information to be easily processed for simple real-time force sensing. A key trend for miniaturization thus involves replacing the camera with distributed photodetectors or color sensors to measure deformation-induced changes in light, creating thinner, more integrable optical sensors [4].

On the other hand, the field of flexible electronics has produced sensors using piezoresistive or capacitive materials with bioinspired microstructures (e.g., porous or pyramid designs) to achieve high sensitivity and conformability on curved surfaces [14,15]. Sensors such as those by Min et al. [2] and Chen et al. [3] use a flexible, multi-layered microstructure with composite material electrodes to measure normal, shear, and torsional forces. These sensors are compact and robust, but they require complex fabrication processes due to the inherent use of microstructures and they also require the use of complex data analysis to extract the force components.

In summary, existing sensors rely on either complex fabrication processes or complex data analysis to extract force components. To surpass these limitations, this paper introduces a novel optical sensing approach engineered for ultra-compact integration. The proposed sensor utilizes a top-layer patterned color surface paired with digital color sensors to function as a visual encoding layer. When forces are applied, they induce measurable, direction-dependent shifts in the reflected color distribution. This design shifts the complexity from convoluted mechanical hardware to optical pattern analysis, enabling the decoupling of normal, shear, and torsional loads within a highly compact 15 × 15 mm^2^ footprint. This work details the sensor’s design, operating principle, and experimental validation through integration with a robotic gripper, demonstrating its capability for closed-loop adaptive force control and stable object manipulation. The proposed sensor presents a scalable, low-profile alternative to conventional multi-axis force sensors, suitable for the next generation of dexterous robotic and haptic systems.

## 2. Materials and Methods

### 2.1. Device Design

**Figure 1:**
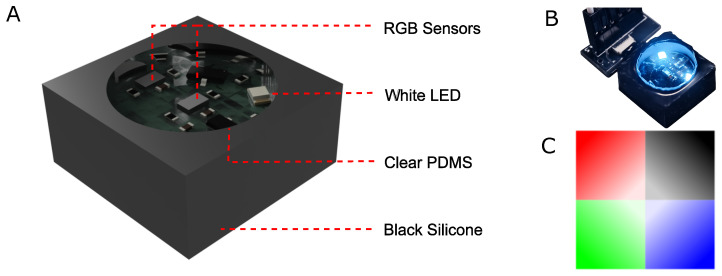
RGB optical force sensor design and components. (**A**) Computer-aided design (CAD) isometric view illustrating the compact 15 × 15 × 6 mm^3^ architecture, featuring a rigid PCB core protected by a dual-layer PDMS and black-tinted silicone encapsulation to minimize lateral light leakage. (**B**) Photograph of the fabricated prototype under illumination; the internal geometry consists of two VEML3328 RGB sensors and two white LEDs arranged to optimize reflected light capture. (**C**) The 4-quadrant visual encoding layer (Red, Green, Blue, and Black) used to translate mechanical deformation into distinct spectral shifts for force vector estimation.

The device is designed around a rigid printed circuit board (PCB) (JiaLiChuang (HongKong) Co., Limited, Shenzhen, China) encased in molded polydimethylsiloxane (PDMS) encapsulation. 2 RGB sensors (VEML3328) (Vishay Intertechnology, Inc., Malvern, PA, USA) are mounted on the PCB, with one at the center of the PCB and the other at the edge. The edge sensor alongside 2 white LEDs form a circular pattern, each 120 degrees apart from each other. The PCB is then encapsulated in 2 steps, first with clear PDMS (Sylgard 184) (The Dow Chemical Company, Midland, MI, USA) and then with black-tinted silicone (Ecoflex 30) (Smooth-On Inc., Macungie, PA, USA). The reason for a two-step encapsulation process is two-fold, one to reduce the amount of light leakage from the side walls of the device, and the other to improve the flexibility of the device such that a higher deformation is achieved with a smaller force, improving sensitivity while maintaining clarity in the optical section of the device. The final thickness of the device is approximately 6 mm, while the overall dimensions are at 15 × 15 × 6 mm^3^. The patterned color surface comprises of quadrants of different colors (red, green, blue and black) which serve as the visual encoding layer. Applied forces cause relative motion or deformation between the top plate and the internal color-patterned layer, altering the reflected light distribution. The color data (R, G, B, Clear) is received by an ESP32-S3 module (Seeed Studio ESP32-S3 XIAO) (Seeed Technology Co., Ltd., Shenzhen, China) and transmitted to a computer for processing. An image of the device is shown in Figure 1.

### 2.2. Device Modelling

In this section, we will describe the theoretical model of the proposed sensor in terms of the plastic deformation of the device under different types of forces as a demonstration of the working principle of the sensor.

#### 2.2.1. Sensor Structure and Operating Principle

The tactile sensor model consists of a rigid color-patterned film, a transparent elastic polydimethylsiloxane (PDMS) layer of thickness *D*, a single light-emitting diode (LED) as a light source, and two spatially separated RGB color sensors with a clear (broadband) channel. One color sensor is located at the center of the printed circuit board (PCB), while the second sensor is placed near the edge. The color-patterned film has a known and fixed spatial color distribution and is assumed to behave as a rigid body under external loading.

When external mechanical stimuli are applied to the sensor surface, the PDMS layer deforms elastically, changing the optical path between the LED, the color-patterned film, and the color sensors below. These changes caused by the deformation result in measurable variations in both the intensity and spectral composition of the reflected light, which are captured by the RGB and clear channels of the sensors. By exploiting the different sensitivities of the clear and RGB channels, as well as the spatial separation between the two sensors, the sensor enables a decoupled estimation of normal force, shear force, and torsional moment.

#### 2.2.2. Optical Measurement Model

For the *i*-th sensor (i=1 for center, i=2 for edge), the sensor outputs four channels: red ($R_{i}$), green ($G_{i}$), blue ($B_{i}$), and clear ($C_{i}$). The clear channel measures the total broadband optical intensity, which can be approximated as the wavelength-integrated intensity of the reflected light:

$$C_{i}=\int_{\lambda_{\mathrm{vis}}}I_{i}\left(\lambda \right)d\lambda$$

The RGB channels measure spectrally weighted components of the same reflected light:

$$\left[\begin{array}{c} R_{i}\\ G_{i}\\ B_{i} \end{array}\right]=C_{i}\cdot c\left(x_{i}^{\prime },y_{i}^{\prime }\right)$$

where c(x,y) is the normalized color vector of the rigid color film at the effective sampling location ($x_{i}^{\prime }$, $y_{i}^{\prime }$), determined by the deformation of the PDMS layer. To decouple spectral information from intensity variations a normalized color vector is defined as

$${\tilde{c}}_{i}=\frac{1}{C_{i}}\left[\begin{array}{c} R_{i}\\ G_{i}\\ B_{i} \end{array}\right].$$

This normalization removes the influence of illumination intensity and optical path length, allowing the RGB channels to encode primarily spatial color information.

#### 2.2.3. Mechanical Deformation of the PDMS Layer

The PDMS layer is modeled as a linear elastic, isotropic material with Young’s Modulus *E* and shear modulus *G*. For small deformations, the following mechanical responses are assumed.

Under an applied normal force $F_{z}$, the PDMS layer undergoes uniform compression:

$$\Delta D=\frac{F_{z}}{k_{z}}\;\mathrm{with}\;k_{z}\approx \frac{EA}{D}$$

where *A* is the effective contact area. The local PDMS thickness becomes:

$$D^{\prime }=D-\Delta D$$

Under a tangential shear force $F_{x}$ or $F_{y}$ (summarized as $F_{s}$), the PDMS layer experiences lateral displacement:

$$\delta =\frac{F_{s}D}{GA}$$

This displacement causes a rigid-body translation of the color-patterned film relative to the sensors.

Under an applied torsional moment $\tau_{z}$, the PDMS layer undergoes angular deformation:

$$\theta =\frac{\tau_{z}D}{GJ}$$

where *J* is the polar moment of the contact area. This results in a rotational motion of the color-patterned film about the sensor’s normal axis.

#### 2.2.4. Force Sensing Mechanism

Normal forces primarily affect the optical path length through the PDMS layer. Compression reduces the distance between the LED, the color film and the sensors, increasing the reflected light intensity. As a result, the clear channel exhibits a strong monotonic increase with normal forces:

$$\Delta C_{i}\propto \Delta D\propto F_{z}$$

Because pure normal compression does not significantly alter the lateral position of the color pattern, the normalized RGB values remain approximately constant. A robust estimator for the normal force is therefore given by

$$F_{z}\propto \frac{C_{1}+C_{2}}{2}$$

which is insensitive to shear and torsional effects.

Shear forces produce a lateral translation of the rigid color film. This translation changes the effective sampling location ($x_{i}^{\prime }$, $y_{i}^{\prime }$) for each sensor, causing distinct color changes that depend on the local gradient of the film. The differential normalized color signal between the two sensors is defined as

$$\Delta {\tilde{c}}_{\mathrm{shear}}={\tilde{c}}_{2}-{\tilde{c}}_{1}$$

For small deformations, this differential signal is approximately proportional to the shear displacement and thus to the applied shear force:

$$\Delta {\tilde{c}}_{\mathrm{shear}}\propto \delta \propto F_{s}$$

The magnitude of this vector encodes the shear force maginitude, while its direction reflects the direction of the applied shear force.

Torsional loading induces a rotation of the color film. Due to its proximity to the rotation center, the central sensor experiences minimal color change, whereas the edge sensor undergoes a significant change in its normalized RGB output. The torsional signal is extracted from the temporal variation in the normalized color vector at the edge sensor:

$$\Delta {\tilde{c}}_{\mathrm{torsion}}={\tilde{c}}_{2}\left(t\right)-{\tilde{c}}_{2}\left(0\right)$$

This variation is proportional to the rotational angle θ, and thus to the applied torsional moment:

$$∥\Delta {\tilde{c}}_{\mathrm{torsion}}∥\;\propto \theta \propto \tau_{z}$$

The sign of the torsional moment can be inferred from the direction of color change along the known color gradients.

Combining the above mechanisms, the sensor response can be approximated by a linearized tactile Jacobian:

$$\left[\begin{array}{c} \Delta C_{\mathrm{avg}}\\ \Delta {\tilde{c}}_{\mathrm{shear}}\\ \Delta {\tilde{c}}_{\mathrm{torsion}} \end{array}\right]=J\left[\begin{array}{c} F_{z}\\ F_{s}\\ \tau_{z} \end{array}\right]$$

where J is approximately diagonal for small deformations, enabling decoupled estimation of normal, shear and torsional forces.

#### 2.2.5. Modeling Assumptions and Validity

This model assumes linear elastic behavior of the PDMS layer, rigid-body motion of the color film, uniform illumination and small deformations. Under these assumptions, the sensor exhibits well-conditioned inverse mapping and robust multi-axis tactile sensing capabilities.

**Figure 2:**
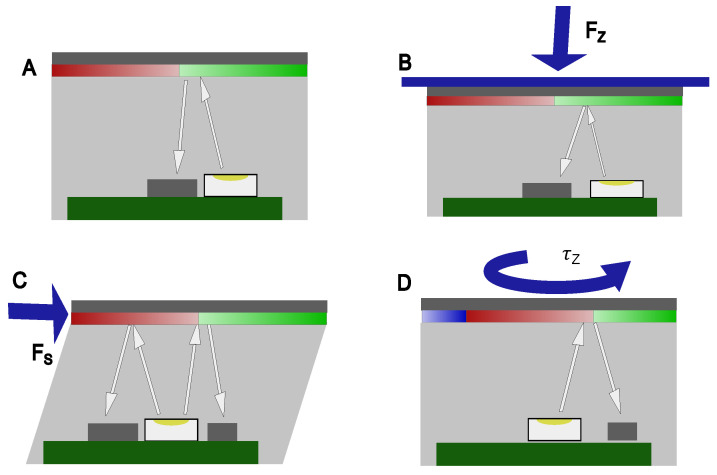
Schematic working principle of the RGB optical force sensor. The device consists of a rigid color-patterned film atop a transparent, elastic polydimethylsiloxane (PDMS) layer, with a central LED and two spatially separated RGB color sensors (one central, one edge-mounted) located on the PCB below. **(A)—Undeformed baseline state ($F_{z}$):** In the absence of external load, the LED illuminates the color-patterned film through the PDMS layer of thickness *D*. The sensors capture a baseline reflected intensity and spectral composition based on the fixed spatial color distribution of the film with the white arrows representing the optical path and the color sections representing the color pattern. **(B)—Normal Force ($F_{z}$):** An applied normal force $F_{z}$ compresses the PDMS layer, reducing its thickness to $D^{\prime }=D-\Delta D$. This reduction in optical path length increases the reflected light intensity captured by the broadband clear channel ($C_{i}$). Because normal compression does not shift the film laterally, the normalized color remains constant; thus, $F_{z}$ is robustly estimated by the average intensity: $F_{z}\propto \frac{C_{1}+C_{2}}{2}$. **(C)—Shear Force ($F_{s}$):** Tangential shear forces ($F_{x},F_{y}$) cause the rigid film to translate laterally by a displacement δ. This translation shifts the effective sampling locations on the color-patterned film, causing a shift in the RGB output. The shear force is calculated using the differential normalized color signal between the two sensors: $\Delta {\tilde{c}}_{\mathrm{shear}}={\tilde{c}}_{2}-{\tilde{c}}_{1}$. **(D)—Torsional Moment ($\tau_{z}$):** Torsional loading induces an angular rotation θ of the color film about the central axis. While the central sensor remains relatively unaffected due to its proximity to the rotation center, the edge sensor experiences a significant change in its normalized color vector. The moment is derived from the temporal variation in the edge sensor’s output: $\Delta {\tilde{c}}_{\mathrm{torsion}}={\tilde{c}}_{2}\left(t\right)-{\tilde{c}}_{2}\left(0\right)$. By combining these distinct optical responses into a linearized tactile Jacobian (J), the system can independently estimate normal, shear, and torsional components even when applied simultaneously.

The overall working principle of the device is illustrated in Figure 2 with a demonstration of the device in Video S1.

### 2.3. Experimental Setup

In order to validate the sensor’s functionality and performance, experiments were conducted using computerized finite-element analysis (FEA) and physical testing. FEA simulations were performed using COMSOL 6.2 Multiphysics software to predict the deformation of the device under different types of forces. Physical testing was carried out using a custom-built robotic gripper integrated with the sensor, applying various forces and torques to evaluate its performance. The results of these experiments are presented in Section 3.

### 2.4. Usage of GenAI

During the preparation of this manuscript, GenAI was used to generate text for the purposes of structuring the paper and consolidating the overall content. GenAI was also used to analyze and summarize common challenges faced by multi-axis tactile sensors and identify methods for overcoming them.

## 3. Results

### 3.1. Sensor Deformation and Stress Simulation

**Figure 3:**
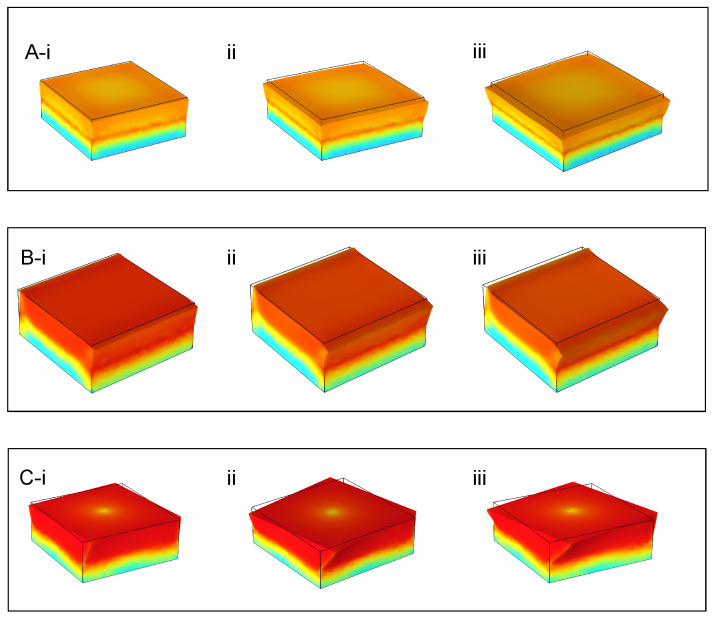
3D COMSOL Multiphysics finite element simulation of the RGB force sensor’s elastomeric structure under normal, shear, and torsion loading. The simulated deformation of the top sensing plate and the corresponding shift in its surface color patterns are illustrated, which form the working principle for optical force transduction. The stress distribution is represented by the colors, increasing from blue to red. (**A**) Application of a normal (vertical) force, showing compressive deformation. Subfigures (**A**) (i–iii) depict increasing magnitudes of normal force, resulting in progressive radial expansion and color gradient shift. (**B**) Application of in-plane shear force. Subfigures (**B**) (i–iii) show increasing shear displacement, leading to a lateral translation and asymmetric tilt of the color pattern. (**C**) Application of torsional (torque) force about the central axis. Subfigures (**C**) (i–iii) illustrate increasing torque, demonstrating a characteristic rotational shear deformation and spiral distortion of the color pattern. For all cases, the simulated change in the reflected light distribution provides a unique, quantifiable signature for each force type and magnitude, enabling multi-axis force discrimination.

To simulate the deformation of the device under different types of forces, FEA simulations were employed utilizing COMSOL Multiphysics software. The model consisted of a hard rectangular solid representing the PCB encased in a 2nd soft shell made of PDMS, with dimensions matching those of the actual prototype. The material properties were set according to the composition of the PDMS used in the encapsulation process. Forces were applied along the normal (vertical), shear (horizontal), and torsion (rotational) axes to observe the resulting deformation across the top surface. The simulation results are shown in Figure 3.

In Figure 3A, the simulation results display a progressive radial expansion of the PDMS substrate under increasing normal forces. The deformation mainly occurs in the upper central region due to the rigid properties of the PCB embedded inside the PDMS block and it gradually increases as the force applied increases from 10 N to 20 N. The stress concentration mainly occurs at the interface between the PCB and the PDMS block as expected and the maximum stress increases from 2.25 MPa at 10 N to 4.50 MPa at 20 N.

In Figure 3B, the simulation shows a lateral translation of the top face with increasing shear forces applied in the situation where the overall force is applied as a linear combination of a compressive normal force and a shear force in order to simulate the force applied by an object held against the sensor. The normal force is held constant at 1 N, while the shear force is varied from 5 N to 15 N. The top face shifts more strongly as the shear force increases from 5 N to 15 N. The simulation also shows an asymmetric tilt of the top face with increasing shear forces applied by a rigid connector. The top face tilts more strongly as the shear force increases from 5 N to 15 N. This shows that the PDMS block starts to behave non-uniformly under large shear forces, which must be accounted for in the calibration and force analysis. The maximum stress in the system occurs at the face that is in the direction of the applied shear aligned with the interface between the top face of the PCB and the PDMS block, increasing from 1.77 MPa at 5 N to 4.88 MPa at 15 N. This shows that the encapsulation of the PCB does affect the deformation and stress concentration of the block under shear forces.

In Figure 3C, the simulation illustrates a gradual rotation of the top face due to the torque moment applied by the rigid connector. The deformation increases as the angle of the applied torque increases from 0.05 radians to 0.25 radians. The simulation starts to break down as the angle of the applied torque increases beyond 0.25 radians, suggesting that the material properties may impose limitations to the maximum torque measureable by the sensor. The maximum stress in this system occurs at the corners of the block aligned with the interface between the top face of the PCB and the PDMS block, increasing from 0.99 MPa at 0.05 radians to 4.98 MPa at 0.25 radians.

### 3.2. Sensor Calibration and Sensitivity Testing

**Figure 4:**
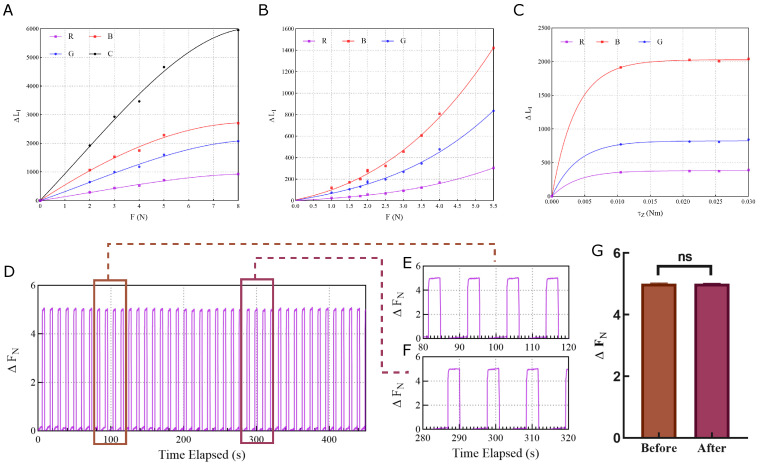
**Static calibration and multi-axis force response of the optical sensor.** (**A**) Normal force calibration showing the change in clear channel intensity ($\Delta L_{C}$) as a function of applied normal force ($F_{z}$). The monotonic increase in intensity reflects the reduction in the optical path length as the PDMS layer undergoes uniform compression, modeled as $\Delta D=\frac{F_{z}}{k_{z}}$. (**B**) Shear force response characterized by the differential normalized color signal ($\Delta {\tilde{c}}_{\mathrm{shear}}$). The lateral displacement of the rigid-body color-patterned film relative to the sensors results in distinct spectral shifts across the R, G, and B channels. (**C**) Torsional moment response derived from the temporal variation in the normalized color vector at the edge sensor ($\Delta {\tilde{c}}_{\mathrm{torsion}}$). Rotational motion of the color film about the sensor’s normal axis induced by torque ($\tau_{z}$) is captured primarily by the edge sensor due to its distance from the rotation center. (**D**) Stability test of sensor measurement under normal force. The sensor is mounted on a robotic gripper and a block was gripped and released repeatedly over a long time. The measured sensor values are converted to force values using the calibration equations. Samples of the measured force values were taken at the beginning (**E**) and end (**F**) of the experiment. (**G**) An unpaired T-test was performed to compare the samples taken from the beginning and end of the experiment. The *p*-value was calculated to be 0.9520, indicating no significant difference in the measured force values.

To calibrate the sensor and assess its sensitivity, a series of experiments were conducted. The sensor was placed on a flat surface, and forces ranging from 0 to 10 N were applied along the normal, shear, and torsion axes using a force sensor. The color data captured by the RGB sensors was processed to extract the force components acting on the sensor. Polynomial regression was utilized to fit the extracted data, yielding equations that relate the force components to the color data. These equations allowed us to convert raw color readings into meaningful force values. The calibration process resulted in polynomial coefficients for each axis, as shown in Figure 4.

In Figure 4A, the calibration curve shows an approximately linear relationship between the change in clear channel intensity ($\Delta L_{C}$) and the applied normal force ($F_{z}$) for small forces ($F_{z}$ < 5 N). The curve shows a positive slope, indicating a reduction in the optical path length as the PDMS layer undergoes uniform compression which is consistent with the model. From a linear interpolation of the calibration curve for the clear channel at small forces, the slope is estimated to be $k_{z}=972.8\;N^{-1}$, indicating a sensitivity of $\frac{1}{972.8}\approx 1.03\times 10^{-3}\;N\;{\mathrm{unit}}^{-1}$. However, the relationship deviates from a straight line for larger forces ($F_{z}$ > 5 N), indicating the presence of nonlinear effects as expected from the simulations.

In Figure 4B, the shear force response shows a linear relationship between the differential normalized color signal ($\Delta {\tilde{c}}_{\mathrm{shear}}$) and the applied shear force ($F_{s}$) for small forces, but the relationship quickly deviates from a straight line for larger forces, again indicating the presence of nonlinear effects. This indicates that the impact of nonlinearity is stronger for shear forces than for normal forces, which is consistent with the simulations. The curve is strictly increasing, indicating an increase in the normalized color signal with increasing shear force in the calibrated direction which is consistent with the model. The slope of the blue curve is estimated to be $k_{s}=126.5\;N^{-1}$ for small forces, indicating a maximum sensitivity of $\frac{1}{126.5}\approx 7.91\times 10^{-3}\;N\;{\mathrm{unit}}^{-1}$.

In Figure 4C, the torque response shows a linear relationship between the temporal variation in the normalized color vector at the edge sensor ($\Delta {\tilde{c}}_{\mathrm{torsion}}$) and the applied torque ($\tau_{z}$) for small torques, but quickly flattens out for larger torques. This indicates that the sensor has limitations in its ability to measure large torques due to material properties, which is consistent with the simulations. The curve is strictly increasing, indicating an increase in the normalized color signal with increasing torque in the calibrated direction which is consistent with the model. The slope of the blue curve for small torques is estimated to be $k_{\tau }=3\times 10^{5}\;{\left(\mathrm{Nm}\right)}^{-1}$, indicating a maximum sensitivity of $\frac{1}{3\times 10^{5}}\approx 3.33\times 10^{-6}\;\mathrm{Nm}\;{\mathrm{unit}}^{-1}$.

A stability test was also performed to validate the sensor’s measurement stability under normal force. The sensor was mounted on a robotic gripper and a block was gripped and released repeatedly over a long time. The measured sensor values were converted to force values using the calibration equations. Samples of the measured force values were taken at the beginning and end of the experiment. An unpaired T-test was performed to compare the samples taken from the beginning and end of the experiment. The *p*-value was calculated to be 0.9520, indicating no significant difference in the measured force values. The results are shown in Figure 4D–G.

**Figure 5:**
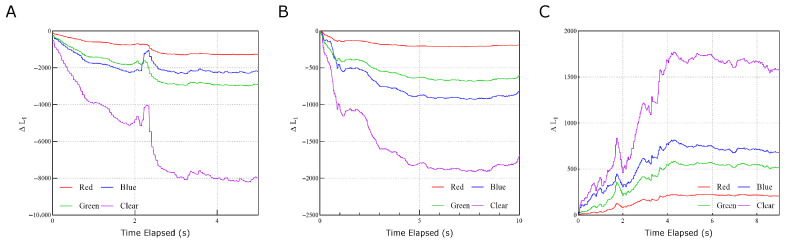
Real-time multi-axis force measurement and decoupling performance. (**A**) Continuous tracking of the clear (C) and RGB channel outputs during dynamic loading cycles. The clear channel data serves as a robust estimator for $F_{z}$ that remains largely insensitive to shear and torsional effects. (**B**) Real-time decoupling of shear force ($F_{s}$) components. The system utilizes the local gradient of the visual encoding layer to translate localized color variations into spatial reconstruction of force magnitude and direction. (**C**) Dynamic response to applied torsional moments. The sign and magnitude of the torsional moment are inferred from the direction of color change along the known color gradients of the 4-quadrant visual encoding layer. These results validate the sensor’s ability to effectively distinguish between different force types using a linearized tactile Jacobian.

Real-time testing was also performed to validate the sensor’s sensing speed and accuracy. The sensor was placed on a flat surface and was contacted with varying forces and torques, and the resulting color data was recorded over time. The data shows that the sensor is responsive and can relay real-time force and torque information with reasonable accuracy, as shown in Figure 5.

In Figure 5A, the clear channel shows the greatest variability with different normal force loads, justifying it as a robust estimator for $F_{z}$. The RGB channels show some variability with different normal force loads, but this can be attributed to the overall brightness of the light source, which can be isolated out by using normalization techniques using the clear channel as a reference.

In Figure 5B, the shear force response shows a much stronger response from the color channels, indicating that the RGB channels can be used to distinguish between shear forces and normal forces. The clear channel here has a weaker response compared to the normal force response, indicating that the clear channel is less sensitive to shear forces. The residual response can be attributed to the slight normal force required to maintain friction between the sensor and the testing surface.

In Figure 5C, the torque response recorded from the edge sensor shows a much stronger response from the color channels, indicating that the RGB channels can be used to distinguish between torsional moments and normal forces. The inversion of the color response from negative to positive is related to the direction of the applied torque, which shows that the color encoding can be used to infer the sign of the applied torque.

### 3.3. Sensor Force Derivation

The applied multi-axis forces are reconstructed from the raw optical data by mapping the measured intensity and spectral shifts back to the mechanical domain using the calibrated tactile Jacobian. As established in the optical measurement model, the clear channel $C_{i}$ serves as the primary indicator for normal forces, while the normalized RGB vectors ${\tilde{c}}_{i}$ provide the spatial encoding for shear and torsion.

The force components are calculated using the following derived system of equations:

$$\left\{\begin{array}{c} F_{z}\approx k\cdot C_{avg}=k\left(\frac{C_{1}+C_{2}}{2}\right)\\ F_{x}=a\Delta R_{1}+b\Delta G_{1}+c\Delta B_{1}\\ F_{y}=d\Delta R_{1}+e\Delta G_{1}+f\Delta B_{1}\\ \tau_{z}=g\Delta R_{2}+h\Delta G_{2}+i\Delta B_{2} \end{array}\right.$$

Following the principle that compression reduces the optical path length and increases reflected intensity, $F_{z}$ is derived from the average of the clear channels $C_{1}$ and $C_{2}$. This averaging minimizes the influence of asymmetric deformations caused by shear or tilt.

The in-plane shear forces $F_{x}$ and $F_{y}$ are calculated as linear combinations of the color deviations ($\Delta L_{I}$) from the central sensor. Because $F_{x}$ and $F_{y}$ induce distinct lateral translations of the 4-quadrant pattern, they produce unique spectral signatures that allow for decoupling directional forces via the coefficients *a* through *f*.

Leveraging the spatial separation of the sensors, $\tau_{z}$ is derived from the edge sensor’s output. While the central sensor remains near the rotation axis and sees minimal color shift, the edge sensor undergoes significant spectral changes proportional to the angular deformation θ.

The constants *k* and *a* through *i* are determined through the polynomial regression of the calibration data, ensuring that the inverse mapping accounts for the specific sensitivities of the VEML3328 sensors and the mechanical properties of the PDMS encapsulation.

### 3.4. Robot Gripper Integration and Testing

To evaluate the proposed RGB optical force sensor’s performance in real-world tactile tasks, it was integrated into a custom-built robotic gripper system mounted on a robotic arm. Communication was established via I2C bus to an ESP32-S3 module, which served as an interface to relay raw spectral data to a host computer through a serial connection for real-time force reconstruction and control.

The experimental protocol focused on three primary dexterous manipulation scenarios: automated grasping, adaptive slip prevention, and human-robot interaction. In the automated grasping task, the gripper was programmed to close until an increase in normal force ($F_{z}$) was detected by the sensor. Upon reaching a predefined force threshold, the control system signaled the gripper to stop closing, ensuring a stable grip while preventing damage to the object.

To demonstrate adaptive control, a “pull force” ($F_{S}$) was applied to the grasped block. The sensor utilized its internal 4-quadrant visual encoding layer to detect the resulting lateral shift in color gradients, identifying the shear force magnitude. In response to this incipient slip condition, the system automatically increased the normal gripping force to enhance frictional contact and maintain object stability. Finally, the sensor’s ability to decouple torsional moments ($\tau_{z}$) was tested. When a user applied a twisting motion to the grasped object, the edge sensor detected the characteristic spectral shifts associated with rotational deformation. This torsional trigger prompted the gripper to release the block, facilitating a seamless hand-over to the user. These results confirm that the sensor provides the high-speed, multi-axis feedback necessary for robust closed-loop tactile control in space-constrained robotic systems.

**Figure 6:**
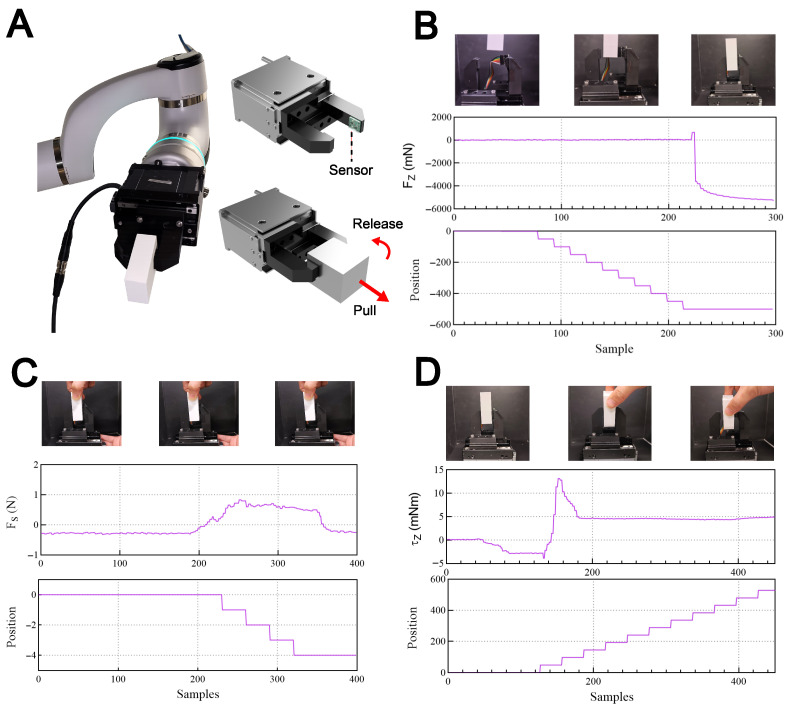
Functional demonstration of the RGB optical force sensor integrated into a robotic gripper. (**A**) Experimental setup showing the sensor mounted on the fingertips of a robotic gripper for multi-axis force perception. (**B**) Automated grasping: the gripper utilizes normal force ($F_{z}$) feedback to detect contact and terminate the closing motion once a stable grip on the block is achieved. (**C**) Adaptive force control: the system detects an external pull force ($F_{S}$) and automatically increases the gripping pressure to prevent slippage by enhancing frictional contact. (**D**) Human-robot interaction: the sensor identifies an applied torsional moment ($\tau_{z}$), triggering an automated release of the object to facilitate a seamless hand-over to a user.

In Figure 6B, the gripper was programmed to close until an increase in normal force ($F_{z}$) of 0.5 N was detected by the sensor. As the required force was detected, the gripper terminates its closing motion as shown in the position graph.

In Figure 6C, a “pull force” ($F_{S}$) was applied to the grasped block, and the sensor detected the resulting lateral shift in color gradients, identifying the shear force magnitude. The gripper responds to the pull force by increasing the normal gripping force to enhance frictional contact and maintain object stability. The gripper stops increasing the force when the pull force is no longer detected.

In Figure 6D, a twisting motion was applied to the grasped block, which is used as a trigger for the gripper to release the block. As a torque of 5 mNm was recorded, the gripper releases the block and opens the gripper fully as shown in the position graph.

## 4. Discussion

The experimental results and finite-element simulations validate the proposed optical sensing paradigm, demonstrating that a compact $15\times 15\;{\mathrm{mm}}^{2}$ footprint can effectively resolve multi-axis force vectors. A central finding is the efficacy of the 4-quadrant visual encoding layer in translating mechanical deformations into unique spectral signatures. As predicted by the optical measurement model, the clear channel exhibits a strong monotonic response to vertical compression due to the reduction in optical path length $D^{\prime }$. This allows the sensor to distinguish normal pressure from tangential loads, which instead produce direction-dependent color shifts via lateral translation.

This optical method provides a localized gradient that encodes the force vector direction using spectral shifting. By using multiple color sensors and the 4-quadrant visual encoding layer, the design achieves high sensitivity and real-time responsiveness within a low-profile 6 mm thickness. The successful integration with a robotic gripper confirms that the sensor can support closed-loop adaptive force control. By providing real-time feedback on shear and torsion, the system can detect incipient slips and adjust gripping force dynamically to ensure stable object manipulation. Future research will focus on extending the derivation equations beyond the linearized Jacobian to account for non-linearities in larger PDMS deformations. Additionally, further optimization of the black-tinted silicone encapsulation will be explored to enhance isolation from ambient light in diverse operational environments or to further enhance sensing by utilizing multiple sensing techniques at the same time such as magnetic sensing by replacing the black dye with magnetic particles. As our design was mainly developed for use in robotic fingertips, currently the device is also limited to single-point force measurement, and future work will focus on extending the sensor to provide multi-point feedback using an array of color sensors and a super-resolution technique to achieve sub-pixel resolution and multi-axis force sensing simultaneously.

## 5. Conclusions

This study introduces a novel optical 3D-force and torsion sensor utilizing a patterned color sensing principle. By employing a dual-layer PDMS encapsulation and a 4-quadrant visual encoding layer, the sensor effectively decouples normal, shear, and torsional loads through differential color analysis. The experimental results and FEA simulations confirm that the device maintains high sensitivity and accuracy across all axes for small forces within a minimal 15 × 15 × 6 mm^3^ footprint. The sensor’s ability to provide real-time feedback for stable object manipulation underscores its potential as a scalable, low-profile alternative for next-generation tactile interfaces in space-constrained robotic systems.

## Data Availability

The data that support the findings of this study are available from the corresponding author upon request.

## Supplementary Materials

The following supporting information can be downloaded at https://www.mdpi.com/article/10.3390/s26051534/s1. Video S1: Force Sensor Live Demonstration.

## Abbreviations

The following abbreviations are used in this manuscript:

CAD	Computer-aided design
CNN	Convolutional neural network
FEA	Finite Element Analysis
FoV	Field of view
KNN	K-Nearest Neighbors
MBTS	Magnetic-based tactile sensors
PCB	Printed circuit board
PDMS	Polydimethylsiloxane
